# Inhibition of VEGF‐dependent angiogenesis and tumor angiogenesis by an optimized antibody targeting CLEC14a

**DOI:** 10.1002/1878-0261.12169

**Published:** 2018-01-26

**Authors:** Taek‐Keun Kim, Chang Sik Park, Jihye Jang, Mi Ra Kim, Hee‐Jun Na, Kangseung Lee, Hyun Jung Kim, Kyun Heo, Byong Chul Yoo, Young‐Myeong Kim, Je‐Wook Lee, Su Jin Kim, Eun Sung Kim, Dae Young Kim, Kiweon Cha, Tae Gyu Lee, Sukmook Lee

**Affiliations:** ^1^ Scripps Korea Antibody Institute Chuncheon South Korea; ^2^ Research Institute National Cancer Center Goyang South Korea; ^3^ Department of Molecular and Cellular Biochemistry Kangwon National University Chuncheon South Korea; ^4^ New Drug Development Center Osong Medical Innovation Foundation South Korea

**Keywords:** angiogenesis, CLEC14a, CTLD, VEGF, tumor angiogenesis

## Abstract

The C‐type lectin‐like domain of CLEC14a (CLEC14a‐C‐type lectin‐like domain [CTLD]) is a key domain that mediates endothelial cell–cell contacts in angiogenesis. However, the role of CLEC14a‐CTLD in pathological angiogenesis has not yet been clearly elucidated. In this study, through complementarity‐determining region grafting, consecutive deglycosylation, and functional isolation, we generated a novel anti‐angiogenic human monoclonal antibody that specifically targets CLEC14a‐CTLD and that shows improved stability and homogeneity relative to the parental antibody. We found that this antibody directly inhibits CLEC14a‐CTLD‐mediated endothelial cell–cell contact and simultaneously downregulates expression of CLEC14a on the surface of endothelial cells. Using various *in vitro* and *in vivo* functional assays, we demonstrated that this antibody effectively suppresses vascular endothelial growth factor (VEGF)‐dependent angiogenesis and tumor angiogenesis of SNU182 human hepatocellular carcinoma, CFPAC‐1 human pancreatic cancer, and U87 human glioma cells. Furthermore, we also found that this antibody significantly inhibits tumor angiogenesis of HCT116 and bevacizumab‐adapted HCT116 human colorectal cancer cells. These findings suggest that antibody targeting of CLEC14a‐CTLD has the potential to suppress VEGF‐dependent angiogenesis and tumor angiogenesis and that CLEC14a‐CTLD may be a novel anti‐angiogenic target for VEGF‐dependent angiogenesis and tumor angiogenesis.

**Abbreviations**
CDR: complementarity‐determining region
CLEC14a: c‐type lectin domain family 14 member
CTLD: c‐type lectin‐like domain
EBM: endothelial basal medium
ECD: extracellular domain
EGM: endothelial growth medium
HUVEC: human umbilical vascular endothelial cell
IgG: immunoglobulin G
VEGF: vascular endothelial growth factor

## 1 Introduction

Monoclonal antibody‐based therapy is an important strategy used to treat diseases, such as tumors, immunological disorders, and eye diseases (Chames *et al*., 2009; Liu *et al*., 2008; Sliwkowski and Mellman, 2013). More than 40 therapeutic antibodies have been approved by the U.S. Food and Drug Administration, and many more are currently being evaluated in clinical trials (Ecker *et al*., 2015). The development of pharmaceutical antibodies is a complex process that requires careful planning and considerable effort. Antibody aggregation and bulk heterogeneous glycosylation of the variable region can adversely affect the quality, safety, and efficacy of pharmaceutical antibodies (Jefferis, 2016; Ratanji *et al*., 2014; Vazquez‐Rey and Lang, 2011; Wright and Morrison, 1993). Thus, it is important to minimize or remove aggregates and glycosylation sites from antibody drug substances and the final pharmaceutical product. However, a poor understanding of the conformational structures of antibodies and limitations in antibody design methods have hindered efforts to improve these attributes of pharmaceutical antibodies.

Angiogenesis is the physiological process through which new blood vessels are grown from pre‐existing vessels, and it is essential for tumor formation, wet age‐related macular degeneration (AMD), and diabetic retinopathy (Adamis *et al*., 1999; Nishida *et al*., 2006). Under pathological conditions, angiogenesis is regulated by a coordinated network of upregulated ligands and receptors (Potente *et al*., 2011), including vascular endothelial growth factor (VEGF), which is a key player in angiogenesis (Hoeben *et al*., 2004). Currently, bevacizumab, a humanized antibody targeting VEGF, is used to treat a variety of cancers and wet AMD (Keating, 2014; Martin *et al*., 2011; Melnikova, 2005). Despite the potential effectiveness of bevacizumab therapy against pathological angiogenesis, however, several side effects have been reported. For example, bevacizumab may adversely affect normal VEGF‐mediated endothelial cell signaling, as the VEGF receptor is significantly expressed in normal endothelial cells; this can lead to various side effects, including bleeding, proteinuria, and gastrointestinal perforation (Gordon and Cunningham, 2005; Taugourdeau‐Raymond *et al*., 2012). In addition, there may be issues with drug resistance to bevacizumab due to the redundancy of various tumor‐secreted angiogenic factors, including placental growth factor, angiopoietin‐2, epidermal growth factor, and VEGF (Giuliano and Pages, 2013; Kopetz *et al*., 2010; Labussiere *et al*., 2016). Thus, the identification of a novel anti‐angiogenic target closely associated with pathological angiogenesis is an important research challenge.

C‐type lectin‐like 14a (CLEC14a) is a type I transmembrane protein that has an extracellular domain (ECD) consisting of a C‐type lectin‐like domain (CTLD), a series of epidermal growth factor‐like domains, and a sushi‐like domain (Rho *et al*., 2011). CLEC14a plays a key role in filopodium formation, endothelial cell–cell contact, endothelial cell migration, and tube formation during angiogenesis (Ki *et al*., 2013; Noy *et al*., 2016; Rho *et al*., 2011). CLEC14a is also a tumor endothelial marker protein that localizes exclusively to tumor vessels, but not normal vessels (Mura *et al*., 2012; Zanivan *et al*., 2013). Through *in vitro* studies, we recently demonstrated that CLEC14a‐CTLD plays a key role in the regulation of angiogenic properties (Ki *et al*., 2013); however, the potential of CLEC14a‐CTLD as an anti‐angiogenic target has yet not been clearly elucidated.

In this study, we optimized this CLEC14a‐CTLD‐targeting antibody to improve its stability and homogeneity. Various *in vitro* and *in vivo* angiogenesis assays showed that antibody‐based modulation of CLEC14a‐CTLD inhibited VEGF‐dependent angiogenesis and tumor angiogenesis by directly inhibiting CTLD‐mediated molecular interactions between CLEC14a molecules and simultaneously downregulating expression of CLEC14a on the surface of endothelial cells. These findings indicate that targeting antibodies to CLEC14a‐CTLD may be a novel and effective strategy to suppress VEGF‐dependent angiogenesis and tumor angiogenesis.

## 2 Materials and methods

### 2.1 Cell culture

All of the cells were maintained at 37 °C with 5% CO_2_ unless otherwise noted. Human umbilical vein endothelial cells (HUVECs; Lonza, Allendale, NJ, USA) were cultured in endothelial growth medium‐2 (EGM‐2; Lonza). SNU182 cells (Korean Cell Bank, Seoul, Korea) were cultured in RPMI 1640 medium (Gibco, Gaithersburg, MD, USA) supplemented with 10% (v/v) fetal bovine serum (FBS; Gibco) and 1% (v/v) penicillin/streptomycin (Gibco). CFPAC‐1 (ATCC, Manassas, VA, USA) and U87 cells (Korea Cell Line Bank, Seoul, Korea) were cultured in Iscove's modified Dulbecco's medium (Gibco) with the same supplements. HCT116 cells (ATCC) were cultured in McCoy's 5a medium (Corning, Steuben County, NY, USA) with 10% FBS (Corning) and 1× antibiotic/antimycotic (Corning). Bevacizumab‐adapted HCT116 cells (HCT116/Beva) (MD Anderson Cancer Center, Houston, TX, USA) were cultured in McCoy's 5a medium with 10% FBS, 1× antibiotic/antimycotic, and 250 μg·mL^−1^ bevacizumab (Genentech/Roche, South San Francisco, CA, USA). HEK293F cells were cultured in Freestyle™ expression medium (Gibco) in a humidified Multitron incubation shaker (Infors HT) with 8% CO_2_.

### 2.2 Generation of complementarity‐determining region‐grafted IgG antibodies

Clones 1–4 were designed to individually graft the six complementarity‐determining regions (CDRs) in the variable heavy (*V*_H_) and light (*V*_L_) chains of the parental antibodies onto each *V*_H_ and *V*_L_ of the four therapeutic antibodies that were selected as framework donors (omalizumab, trastuzumab, adalimumab, and bevacizumab). Then, each set of CDR‐grafted *V*_H_ and *V*_L_ was synthesized (Integrated DNA Technologies). Each *V*_H_ was designed to incorporate *Eco*RI and *Apa*I restriction sites at the 5′ and 3′ ends, respectively, and each *V*_L_ was designed to incorporate *Hin*dIII and *Bsi*WI restriction sites at the 5′ and 3′ ends, respectively. Each set of synthesized *V*_H_ and *V*_L_ oligonucleotides was cloned into the bicistronic mammalian expression vector pcDNA3.1 (Invitrogen, Carlsbad, CA, USA), which encodes the human IgG1 CH1/hinge/CH2/CH3 domains in the *V*_H_ cloning site and the constant kappa chain in the *V*_L_ cloning site (Sambrook and Russell, 2001). Four sets of CDR‐grafted IgG antibodies (Clone 1‐4 IgGs) were produced in HEK293F cells and purified as described previously (Ki *et al*., 2013). Protein concentrations were quantified using a NanoDrop 2000 spectrophotometer (Thermo Fisher Scientific, Waltham, MA, USA), and antibodies were dialyzed against phosphate‐buffered saline (PBS). Sample purity was analyzed by SDS/polyacrylamide gel electrophoresis (PAGE) and Coomassie brilliant blue staining. The final pooled fractions were aliquoted and stored at −80 °C.

### 2.3 *In silico* stability analysis of four CDR‐grafted IgG antibodies

Developability index (DI) predictions were performed as described previously with minor modifications (Lauer *et al*., 2012). The DI rankings of the four CDR‐grafted IgG antibodies were calculated using computational modeling in discovery studio v3.5 (Accelrys Software Inc., Burlington, MA, USA). The structures of the four CDR‐grafted IgG antibodies (Clone 1–4 IgGs) were first generated using the full‐length antibody models (build model for IgG_1_) and antibody loop models (obtained by analyzing and refining the model; discovery studio v3.5). The crystal structure of a human IgG1 molecule (PDB: 1hzh) was used as the template. The most stable antibody structure was selected, and each CDR‐grafted antibody model was produced using a DI protocol.

### 2.4 Assessment of antibody aggregation

After purification, the absorbance of each antibody sample in PBS was measured at 280 and 340 nm using a UV/visible spectrophotometer (Ultrospec 2100 Pro; GE Healthcare, Broomfield, CO, USA) in a cuvette with a 10‐mm path length. The aggregation index was calculated from the UV absorbance using the following equation as described previously (Paul *et al*., 2015): 100 × (Abs_340_/[Abs_280_–Abs_340_]).

### 2.5 Generation and isolation of four deglycosylated IgGs

To remove the predicted *N*‐glycosylation site within the light‐chain CDR1 of Clone 1 IgG, a semisynthetic single‐chain variable fragment (scFv) library was generated by random mutation with trinucleotide NNK oligonucleotides combined with overlap extension PCR as described previously (Barbas, 2001). Following two sequential steps to preclear Fc binders from the library, three rounds of biopanning were performed with magnetic beads coated with hCLEC14a‐CTLD‐Fc or mCLEC14a‐CTLD‐Fc (4 μg) using phage display technology (Ki *et al*., 2013). Ninety‐six phage clones were randomly selected from colonies grown on output plates and tested for reactivity to human and mouse CTLDs using phage enzyme immunoassays. After sequencing the DNAs of the final scFv clones, four scFv clones with different sequences were randomly selected and converted to IgG antibodies.

### 2.6 Enzyme‐linked immunosorbent assay (ELISA)

To determine the reactivity of CLEC14a‐CTLD IgGs to human and mouse CLEC14a‐CTLDs, the wells of 96‐well plates were coated with hCLEC14a‐CTLD‐Fc, mCLEC14a‐CTLD‐Fc, or Fc (0.1 μg) in PBS. The plates were incubated overnight at 37 °C, washed three times with PBS containing 0.05% (v/v) Tween 20 (PBS‐T), and incubated with 3% (w/v) bovine serum albumin (BSA) in PBS‐T (blocking buffer) for 1 h at 37 °C. Then, the plates were incubated with 1 μg of Clone 1 IgG or deglycosylated CLEC14a‐CTLD IgGs (deglyco C1‐C4 IgGs) in blocking buffer for 2 h at 37 °C. Following two washes with PBS‐T, horseradish peroxidase (HRP)‐conjugated anti‐human lambda light‐chain antibody (1 : 1000; Bethyl Laboratories, Montgomery, TX, USA) in blocking buffer was added, and the plates were incubated for 1 h at 37 °C. To perform a competition assay with deglyco C1 IgGs, 0.625 μg of hCLEC14a‐ECD was coated onto each well of a 96‐well plate, and the plate was treated with blocking buffer for 2 h at 37 °C. Then, 1 μg of hCLEC14a‐CTLD‐Fc‐HRP was incubated with hCLEC14a‐ECD in the presence or absence of increasing concentrations of deglyco C1 IgGs for 2 h at room temperature. To determine the binding region of deglyco C1 IgG to CLEC14a‐CTLD, 1 μg of Fc, CTLD(1‐142)‐Fc, CTLD(1‐42)‐Fc, or CTLD(122‐142)‐Fc were coated onto each well of a 96‐well plate, and the plate was treated with blocking buffer for 2 h at 37 °C. Following incubation with 1 μg of parental IgG, or deglyco C1 IgG, and/or bevacizumab for 1 h at 37 °C, HRP‐conjugated anti‐human lambda light‐chain antibody (1 : 1000; Bethyl Laboratories) and anti‐human Fab antibody (1 : 5000; Thermo Fisher Scientific) were added to wells, and the plates were incubated for 2 h at room temperature. Following three washes with PBS‐T, 100 μL of 3,3′,5,5′‐tetramethylbenzidine substrate solution (TMB; BD Biosciences, San Jose, CA, USA) was added to each well. The reactions were stopped by the addition of an equal volume of 1 N H_2_SO_4_. Optical density was measured at 450 nm using a microplate reader (VICTOR X4; PerkinElmer, Boston, MA, USA).

### 2.7 Flow cytometry

To investigate the specific binding of deglyco C1 IgG to HUVECs, 2 × 10^5^ cells were fixed with 4% (w/v) paraformaldehyde (PFA) for 20 min at room temperature. After blocking with PBS containing 1% (w/v) BSA for 1 h at room temperature, the cells were incubated with deglyco C1 IgG (20 μg·mL^−1^) for 2 h at room temperature. Then, the cells were washed and incubated with FITC‐conjugated anti‐human IgG (Fab specific; 1 : 500; Sigma‐Aldrich, St. Louis, MO, USA) for 1 h at room temperature. To determine whether deglyco C1 IgG downregulated CLEC14a, 3 × 10^5^ HUVECs were incubated in the presence or absence of deglyco C1 IgG (20 μg·mL^−1^), fixed with 4% (w/v) PFA, washed, and incubated with sheep anti‐CLEC14a polyclonal antibody (7.5 μg·mL^−1^; R&D Systems, Minneapolis, MN, USA) followed by incubation with Northern Lights 493 Fluorochrome (NL493)‐labeled anti‐sheep antibody (1 : 200; R&D Systems). The effects of deglyco C1 IgG on endothelial cell activation were evaluated by incubating 2 × 10^5^ HUVECs in the presence or absence of 20 ng·mL^−1^ human tumor necrosis factor‐α (Millipore) or deglyco C1 IgG (20 μg·mL^−1^) for 24 h. After blocking with PBS containing 1% (w/v) BSA for 1 h at room temperature, cells were incubated with 2 μg per well anti‐ICAM‐1 or anti‐VCAM‐1 antibody for 1 h at 37 °C and then with Alexa Fluor 488‐conjugated anti‐rabbit IgG (1 : 1000; Invitrogen) for 1 h at 37 °C. The samples were analyzed using flow cytometry (BD FACSCalibur; BD Biosciences) with the aid of flowjo software (TreeStar, Ashland, OR, USA).

### 2.8 Real‐time measurement of antibody–antigen interactions

A biolayer interferometry assay was performed using an Octet^®^ RED96 system (ForteBio, Pall Life Sciences, Westborough, MA, USA) as described previously (Park *et al*., 2016). Briefly, a total of 1 μg of hCLEC14‐ECD was immobilized onto amine‐reactive biosensors. Deglyco C1 IgG was twofold serially diluted (62.5–7.8 nm) in 1× kinetics buffer. Association and dissociation rate constants were determined by fitting the data to a 1 : 1 binding model using fortebio octet data analysis software version 7.1.

### 2.9 Tube formation assay

Forty‐eight‐well plates were coated with 150 μL of Matrigel (Corning) and incubated for 30 min at 37 °C. To investigate the effects of CLEC14a‐CTLD IgGs on tube formation, 1 × 10^5^ HUVECs that had been cultured in EGM‐2 were seeded onto Matrigel‐coated plates and incubated in the presence or absence of Clone 1 IgG, deglycosylated CLEC14a‐CTLD IgGs, or bevacizumab (20 μg·mL^−1^) for 18 h at 37 °C. To evaluate the effect of deglyco C1 IgG on VEGF‐induced tube formation, 1 × 10^5^ HUVECs that had been cultured in endothelial cell basal medium (EBM) containing 20 ng·mL^−1^ recombinant human VEGF (rhVEGF) were seeded onto Matrigel‐coated plates and incubated in the presence of 20 μg·mL^−1^ deglyco C1 IgG for 18 h at 37 °C. Images were obtained using a light microscope (Leica DM IL LED, Heidelberg, Nussloch, Germany), and tube formation was quantified by counting the total number of tube branches.

### 2.10 Migration assay

A CytoSelect 24‐well wound‐healing assay kit (Cell Biolabs Inc., San Diego, CA, USA) was used according to the manufacturer's instructions. Briefly, 3 × 10^6^ HUVECs were added to each well of the wound‐healing insert and grown to confluence. Then, the inserts were removed from the wells, and the cells were washed twice with PBS. The HUVECs were incubated in the absence or presence of Clone 1 IgG or deglyco C1 IgG (20 μg·mL^−1^) for 8 h at 37 °C. After staining with crystal violet (Sigma), images were acquired using a light microscope (Leica DM IL LED), and migration distances were measured manually.

### 2.11 Measurement of CLEC14a‐mediated cell–cell contact

CLEC14a‐mediated cell–cell contact assays were performed as described previously (Ki *et al*., 2013). Briefly, 1.5 × 10^7^ HEK293F cells in suspension were transfected with plasmids encoding wild‐type CLEC14a (WT‐CLEC14a), cultured overnight in Freestyle 293 expression medium, and seeded in 6‐well plates (5 × 10^5^ cells per well). The cells were maintained in the absence or presence of deglyco C1 IgG (20 μg·mL^−1^) for 8 h. Cell aggregates (masses with > four cells) were counted in at least 10 visual fields using a light microscope (Leica DM IL LED).

### 2.12 Cell ELISA

Human umbilical vein endothelial cells (1 × 10^4^) were grown in 0.1% (w/v) gelatin‐coated wells of a 96‐well plate overnight at 37 °C. Following fixation with 4% (w/v) PFA, the cells were incubated with 3 μg·mL^−1^ hCLEC14a‐CTLD‐Fc‐HRP in the presence or absence of increasing concentrations of 20 μg·mL^−1^ deglyco C1 IgG for 2 h at 37 °C. After three washes with ice‐cold PBS, 100 μL of TMB substrate solution (BD Biosciences) was added to each well. The reaction was stopped by the addition of an equal volume of 1 N H_2_SO_4_. The optical density was measured at 450 nm using a spectrophotometer (VICTORTM™ X4; PerkinElmer).

### 2.13 Cell viability assay

Human umbilical vein endothelial cells (5 × 10^3^) were placed into 0.1% (w/v) gelatin‐coated wells of a 96‐well plate and incubated in EGM‐2 in the presence or absence of 20 μg·mL^−1^ deglyco C1 IgG or 36 μg·mL^−1^ 5‐fluorouracil for 48 h at 37 °C. Cell viability was determined using the Cell Counting Kit‐8 (Dojindo Laboratories, Rockville, MD, USA) according to the manufacturer's instructions. The final optical density was measured at 450 nm using a spectrophotometer (VICTORTM™ X4).

### 2.14 Immunocytochemistry

Human umbilical vein endothelial cells (5 × 10^4^) that were grown on 0.1% (w/v) gelatin‐coated glass coverslips (Marienfeld‐Superior, Paul Marienfeld GmbH & Co. KG, Lauda‐Königshofen, Germany) were incubated in the presence or absence of 20 μg·mL^−1^ deglyco C1 IgG for 24 h at 37 °C. The cells were fixed in 4% (w/v) PFA, blocked with PBS containing 5% (w/v) BSA and 0.1% (v/v) Triton X‐100 for 1 h at 37 °C, and then incubated with one unit/well rhodamine–phalloidin (Molecular Probes) and 0.1 μg·mL^−1^ Hoechst 33258 (Sigma‐Aldrich) for 1 h at room temperature. Images were acquired with a FluoView FV300 confocal microscope (Olympus, Cypress, CA, USA).

### 2.15 *In vivo* toxicity testing

Seven‐week‐old female BALB/c‐nude mice (*n* = 4) were injected intravenously twice a week with 10 mg·kg^−1^ of deglyco C1 IgG, and their body weights were measured on day 1 and day 28 after antibody injection. After 30 days, the animals were sacrificed and blood samples were collected. The blood samples were centrifuged at 7000 r.p.m. for 20 min at 4 °C, and sera were stored at −80 °C. Serum glutamic oxaloacetic transaminase (GOT), glutamic pyruvic transaminase (GPT), blood urea nitrogen (BUN), creatinine (CRE), and total bilirubin (TBIL) levels were measured using a Fuji Dri‐Chem 3500 biochemistry analyzer (Fujifilm, Tokyo, Japan). TUNEL staining was performed as described previously (Heo *et al*., 2016).

### 2.16 Immunoblot analysis

To evaluate the influence of deglyco C1 IgG on VEGF signaling, 10 μg of cell lysates from VEGF‐treated HUVECs grown in the presence or absence of 20 μg·mL^−1^ deglyco C1 IgG was separated by SDS/PAGE and transferred onto nitrocellulose membranes. After blocking in Tris‐buffered saline and Tween 20 buffer (TBS‐T; 10 mm Tris/HCl pH 7.5, 150 mm NaCl, and 0.05% [v/v] Tween 20) containing 5% (w/v) skim milk, the membranes were incubated with rabbit anti‐VEGFR2 (1 : 1000; Cell Signaling Technology, Danvers, MA, USA), anti‐pVEGFR2 (Tyr1175; 1 : 1000; Cell Signaling Technology), anti‐Akt (1 : 1000; Cell Signaling Technology), anti‐pAkt (Ser473; 1 : 1000; Cell Signaling Technology), anti‐ERK (1 : 1000; Cell Signaling Technology), or anti‐pERK (Thr202/Tyr204; 1 : 1000; Cell Signaling Technology) antibodies overnight at 4 °C. After several washes with TBS‐T, membranes were incubated with the species‐appropriate HRP‐conjugated secondary antibody. Following several washes with TBS‐T, protein bands were visualized using SuperSignal West Pico Chemiluminescent Substrate (Pierce) according to the manufacturer's instructions.

### 2.17 Aortic ring sprouting assay

Aortic rings were obtained from 7‐week‐old Sprague Dawley rats (NARA Biotech, Seoul, Korea). The aortas were cut into 1‐mm segments. Individual segments were embedded in 40 μL of Matrigel (Corning) in 96‐well tissue culture plates and incubated for 30 min at 37 °C in 5% CO_2_. Then, the segments were covered with an additional 40 μL of Matrigel in the presence of EBM, EBM containing 20 ng·mL^−1^ of rhVEGF, or EBM containing rhVEGF and deglyco C1 IgG or bevacizumab (20 μg·mL^−1^). The samples were then incubated for 6 days at 37 °C. Images were captured using a light microscope (Leica DM IL LED), and the numbers of vessels sprouting from each aortic ring were counted manually.

### 2.18 *In vivo* angiogenesis assay

All of the experiments related to VEGF‐dependent angiogenesis or SNU182, CFPAC‐1, and U87 cell‐derived tumor angiogenesis were performed following the protocol approved by the Institutional Animal Ethics Committee of the Kangwon National University (accreditation number KW‐151028) and in accordance with IACUC guidelines. To investigate the effect of deglyco C1 IgG on VEGF‐dependent angiogenesis, 0.5 mL of Matrigel (Corning) containing 5 × 10^6^ HUVECs and 100 ng of rhVEGF in the presence or absence of a single 10 mg·kg^−1^ dose of deglyco C1 IgG or bevacizumab was subcutaneously injected into both flank regions of 4‐week‐old BALB/c male nude mice (NARA Biotech). To investigate the effect of deglyco C1 IgG on tumor angiogenesis, 0.5 mL of Matrigel containing 5 × 10^6^ SNU182, CFPAC‐1, or U87 cells in the presence or absence of a single 10 mg·kg^−1^ dose of deglyco C1 IgG or bevacizumab was subcutaneously injected into both flank regions of 4‐week‐old BALB/c male nude mice (NARA Biotech). After 14 days, the mice were anesthetized, and the Matrigel plugs were removed without removing adjacent connective tissue. The total hemoglobin content in the plugs was quantified using Drabkin's Reagent Kit 525 (Sigma‐Aldrich). All of the experiments related to HCT116 and HCT116/Beva cell‐derived tumor angiogenesis were conducted in accordance with the policies of the KBio Institutional Animal Care and Use Committee (KBIO‐IACUC‐2016‐85). Six‐week‐old NCr female nude mice (Koatech Co., Pyeongtaek, Korea) were subcutaneously injected in the ventral region with 0.5 mL of Matrigel (Corning) containing 1 × 10^6^ HCT116 or HCT116/Beva with a single 5 mg·kg^−1^ dose of deglyco C1 IgG or bevacizumab. After 11 days, the plugs were carefully removed, photographed, and embedded in optimal cutting temperature (OCT) compound (Sakura, Torrance, CA, USA) for assessment of microvessel density by immunohistochemical staining.

### 2.19 Immunohistochemistry

The OCT‐embedded tumor specimens were cut into 5‐μm‐thick sections using a cryostat microtome (CM 1950; Leica) for immunohistochemical analysis. The sections were blocked with 3% BSA in PBS for 1 h, incubated with a rat anti‐mouse primary CD31 antibody (BD Biosciences) for 24 h at 4 °C, and then incubated with an Alexa 647‐conjugated goat anti‐rat IgG secondary antibody (Invitrogen) for 1 h at room temperature in the dark. After washing with PBS, the sections were stained with 4′, 6‐diamidino‐2‐phenylindole, dihydrochloride (Sigma‐Aldrich) for 5 min at room temperature. Fluorescence images were collected using a confocal laser scanning microscope (LSM700; Carl Zeiss, Oberkochen, Germany), and microvessel density was determined by quantifying CD31‐positive pixels in digitized images with LSM 700 zen software (Carl Zeiss). To detect *in vivo* apoptotic activity, U87‐derived tumors treated with or without a single 10 mg·kg^−1^ dose of deglyco C1 IgG or bevacizumab for 2 weeks were mounted on slides and fixed with 4%(w/v) PFA. Staining was then performed using a terminal deoxynucleotidyl transferase‐mediated dUTP‐biotin nick end labeling (TUNEL) assay kit (*In situ* cell death detection kit; Roche) according to manufacturer's instruction. The apoptotic indices were determined by counting the numbers of positive cells from four randomly selected high power fields.

### 2.20 Two‐dimensional gel electrophoresis

Fifteen micrograms of each protein sample were applied to 13‐cm immobilized pH 3–10 nonlinear gradient strips, focused at 8000 V within 3 h, and separated on 10% polyacrylamide gels (Bio‐Rad, Hercules, CA, USA). Two‐dimensional gels were stained with colloidal Coomassie blue (Invitrogen) for 24 h and then destained with deionized water.

### 2.21 Statistical analysis

Data were analyzed with GraphPad Prism 5.0 (graphpad Software, La Jolla, CA, USA) using the two‐tailed Student's *t*‐test for comparisons between two groups and one‐way ANOVA with Bonferroni's correction for multiple comparisons. All of the data were presented as mean ± SEM. *P* values < 0.05 were considered statistically significant.

## 3 Results

### 3.1 Generation of an optimized antibody via CDR grafting

**Figure 1:**
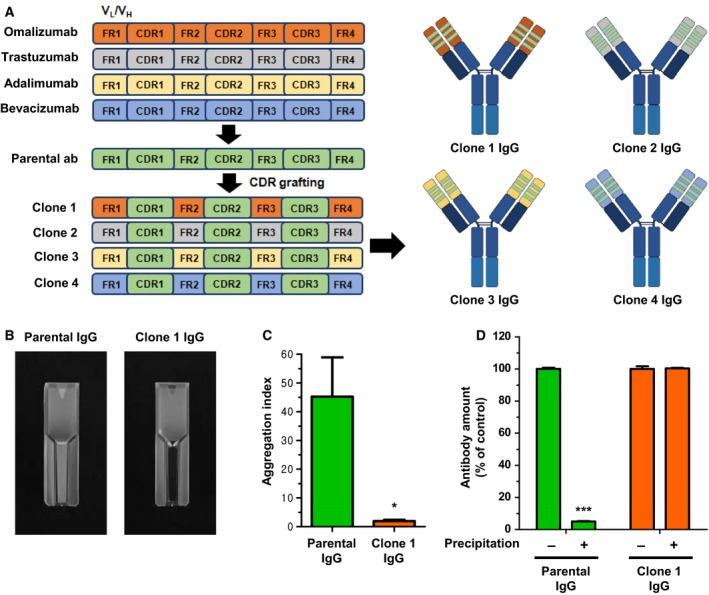
Generation of an optimized antibody with improved stability via CDR grafting. (A) Schematic illustration of the CDR grafting of the parental IgG into omalizumab, trastuzumab, adalimumab, or bevacizumab. (B) Visual observation of antibody aggregation in preparations of the parental IgG and Clone 1 IgG. (C) The aggregation indices of parental IgG and Clone 1 IgG were measured spectrophotometrically. (D) Quantification of parental IgG and Clone 1 IgG antibodies before and after antibody precipitation. All of the values represent the mean ± SEM of triplicate measurements from two independent experiments. *P < 0.05 and ***P < 0.001.

We previously reported that the parental antibody to CLEC14a‐CTLD specifically regulated angiogenic properties *in vitro* (Ki *et al*., 2013). However, this antibody displayed substantial aggregation during antibody purification, and thus, required further optimization to improve the aggregation problem. Therefore, in this study, we performed CDR grafting. Six CDRs of the parental antibody were grafted onto the *V*_H_ and *V*_L_ chains of four therapeutic antibodies: omalizumab, trastuzumab, adalimumab, and bevacizumab (Fig. 1A). We then calculated DIs for these CDR‐grafted IgG antibodies (Clone 1 to Clone 4 IgGs). The DI is a rapid predictive index used to rank monoclonal antibodies based on their aggregation propensities; a higher DI indicates a higher aggregation propensity. The DIs of the CDR‐grafted IgG antibodies were individually compared with the DIs of the parental IgGs using *in silico* analysis. Clone 1 IgG, which was derived from omalizumab, exhibited a lower DI than its parental IgG and clones 2–4 (Table S1); thus, Clone 1 exhibited the highest stability.

To verify these findings, we compared the aggregation propensities of the parental IgG and Clone 1 IgG by visual observation and spectrophotometry. Prominent aggregation was observed visually only for the parental IgG (Fig. 1B). Spectrophotometric measurements revealed that the aggregation indices of the parental and Clone 1 IgGs were approximately 23.4 and 1.5, respectively, indicating that Clone 1 IgG is more soluble than the parental IgG (Fig. 1C). Precipitation and centrifugation‐based removal of aggregates recovered approximately 5% of the parental IgGs, whereas no aggregation was detected for Clone 1 IgG (Fig. 1D). Collectively, these results suggest that CDR grafting and computational modeling may be an effective strategy for designing a therapeutic antibody platform with improved stability and that we successfully generated a more stable version of our antibody.

### 3.2 Optimized antibody selection by consecutive deglycosylation and functional isolation

**Figure 2:**
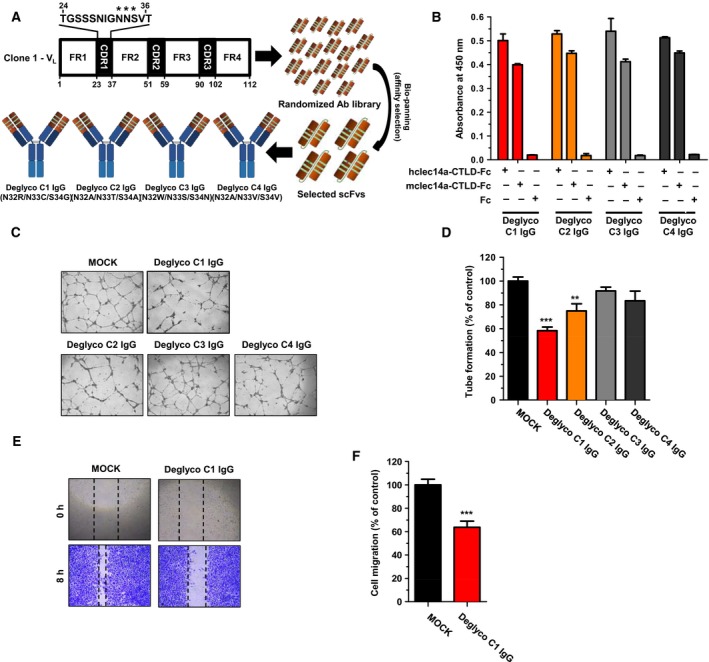
Selection of an optimized lead antibody via consecutive deglycosylation and functional isolation. (A) Schematic illustration of antibody deglycosylation and the phage display technique used to select four deglycosylated IgG clones (deglyco C1‐C4 IgGs). (B) The binding specificities of the four deglycosylated IgG clones to human CTLD‐Fc, mouse CTLD‐Fc, and Fc alone were measured by ELISA. (C) HUVEC tube formation was assessed, and an optimized lead antibody that inhibited CLEC14a‐mediated angiogenesis was selected. Clone 1 IgG was used as the positive control. (D) The total number of tube branches formed was expressed as a percent of tube formation by the control (MOCK). (E) A wound‐healing assay was performed to measure the effect of deglyco C1 IgG on endothelial cell migration. (F) The extent of closure at the wound margins was expressed as a percent of the migration of the control (MOCK). (F) Motility analyses of Clone 1 and deglyco C1 IgG were performed using one‐dimensional gel electrophoresis under reducing conditions. All of the values represent the mean ± SEM of triplicate measurements from two independent experiments. **P <* *0.01 and ***P <* *0.001.

Heterogeneity of glycosylation may affect the quality of critical attributes during the development and production of therapeutic proteins (Sola and Griebenow, 2010). We assessed computationally the potential glycosylation sites of Clone 1 IgG and identified a putative *N*‐glycosylation site within light‐chain CDR1 (data not shown). To remove this putative *N*‐glycosylation site, we generated a semisynthetic antibody library with random mutations at the predicted *N*‐glycosylation site. We then performed alternate biopanning using phage display technology with magnetic bead‐coated Fc fusions of human and mouse CLEC14a‐CTLD (hCLEC14a‐CTLD‐Fc and mCLEC14a‐CTLD‐Fc) and selected four clones (deglyco C1‐C4) that were highly reactive and had different amino acid sequences at the predicted *N*‐glycosylation site (Fig. 2A). These scFv clones were converted to IgGs, expressed in human embryonic kidney (HEK) 293 cells, and purified. ELISA revealed that deglyco C1‐C4 IgGs specifically bound to human and mouse CLEC14a‐CTLD (Fig. 2B).

To isolate a lead antibody that inhibits CLEC14a‐mediated angiogenesis, we evaluated HUVEC tube formation and migration in the presence or absence of Clone 1 IgG and deglyco C1–C4 IgGs. We found that deglyco C1 IgG exhibited the most potent inhibitory effects on HUVEC tube formation (Fig. 2C,D) and migration (Fig. 2E,F) compared with the other three deglycosylated IgGs suggesting that deglyco C1 IgG should be considered the lead antibody among the tested candidates. We also verified that the desired changes had been successfully made in the deglyco C1 IgG light‐chain CDR1 (N32R, N33C, and S34G).

Next, we analyzed the biochemical properties of deglyco C1 IgG. One‐dimensional electrophoresis under reducing conditions revealed that the molecular mass of deglyco C1 *V*_L_ was similar to the predicted molecular mass of approximately 25 kDa, whereas the molecular mass of Clone 1 *V*_L_ (the glycosylated form) was much higher (Fig. 2G). Two‐dimensional electrophoresis revealed that the heterogeneous pattern observed in the low isoelectric point (pI) range of Clone 1 scFv was absent from deglyco C1 scFv, indicating that deglyco C1 IgG exhibited improved homogeneity (Fig. S1). Furthermore, we confirmed the potent binding of deglyco C1 IgG onto the surface of HUVECs (Fig. S2A). We also performed real‐time measurements of antibody–antigen interactions and found that the equilibrium dissociation constant (*K*_D_) of deglyco C1 IgG for the ECD of hCLEC14a (hCLEC14a‐ECD) was approximately 6 nm (Fig. S2B). Collectively, these results suggest that the selected lead antibody is a homogeneity‐improved anti‐angiogenic antibody that could potentially suppress angiogenesis *in vivo*.

### 3.3 Elucidation of the mode of action of the optimized lead antibody

**Figure 3:**
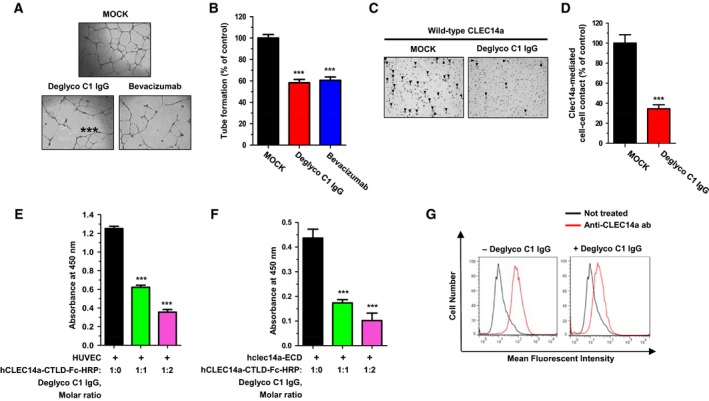
Elucidation of the mechanism of action of the optimized lead antibody. (A) Images depicting tube formation by HUVECs in the absence (MOCK) or presence of 20 μg·mL^−1^ deglyco C1 IgG or bevacizumab as the positive control. Images were captured at 18 h. (B) The number of total branches in the absence (MOCK) or presence of 20 μg·mL^−1^ deglyco C1 IgG or bevacizumab was expressed as a percent of tube formation in the control (MOCK). (C) HEK293F cells transfected with wild‐type CLEC14a were incubated in the absence (MOCK) or presence of deglyco C1 IgG for 6 h. Cell aggregates (aggregate mass > 4 cells; arrowheads) were counted under a light microscope. (D) The number of aggregates per field was expressed as a percent of the control (MOCK). (E) HUVECs were coated onto the wells of a microtiter plate, and binding of hCLEC14a‐(CTLD)‐Fc‐HRP to the HUVECs in the presence or absence of increasing concentrations of deglyco C1 IgG was measured by ELISA. (F) hCLEC14a‐ECD was coated onto the wells of a microtiter plate and binding of hCLEC14a‐CTLD‐Fc‐HRP to hCLEC14a‐ECD in the presence or absence of increasing concentrations of deglyco C1 IgG was measured by ELISA. (G) HUVECs that were incubated in the presence or absence of deglyco C1 IgG were fixed, stained with anti‐CLEC14a antibody, and analyzed by flow cytometry. All of the values represent the mean ± SEM of triplicate measurements from three independent experiments. ***P <* *0.001.

We first compared the effect of deglyco C1 IgG and bevacizumab (as a positive control) on tube formation. We found that deglyco C1 IgG and bevacizumab inhibited tube formation similarly and significantly (Fig. 3A,B). To investigate the mechanism of action of deglyco C1 IgG in angiogenesis, we performed functional assays of CLEC14a‐mediated cell–cell contact. HEK293F cells were transfected with wild‐type CLEC14a, and cell aggregate formation was observed in the presence or absence of deglyco C1 IgG. Our results revealed that deglyco C1 IgG suppressed strongly aggregation of HEK293F cells that were transfected with wild‐type CLEC14a (Fig. 3C,D). These results suggest that deglyco C1 IgG may inhibit strongly CLEC14a‐mediated endothelial cell–cell contact during angiogenesis.

To analyze the molecular mechanism of deglyco C1 IgG in angiogenesis, we treated HUVECs with HRP‐conjugated hCLEC14a‐CTLD‐Fc (hCLEC14a‐CTLD‐Fc‐HRP) in the presence or absence of increasing concentrations of deglyco C1 IgG. We found that deglyco C1 IgG inhibited significantly the binding of CLEC14a‐CTLD to HUVECs in a concentration‐dependent manner (Fig. 3E). We also incubated hCLEC14a‐CTLD‐Fc‐HRP with purified hCLEC14a‐ECD in the presence or absence of increasing concentrations of deglyco C1 IgG. Our results revealed that deglyco C1 IgG inhibited directly the molecular interactions between hCLEC14a‐CTLD and hCLEC14a‐ECD in a concentration‐dependent manner (Fig. 3F). Furthermore, using flow cytometry, we confirmed that deglyco C1 IgG downregulated expression of CLEC14a on the surface of HUVECs (Fig. 3G). Collectively, these results suggest that deglyco C1 IgG specifically inhibits CLEC14a‐CTLD‐mediated molecular interactions between adjacent CLEC14a molecules and concomitantly downregulates CLEC14a expression on endothelial cells during angiogenesis.

### 3.4 Effects of the optimized lead antibody on the viability, morphology, and activation of endothelial cells

**Figure 4:**
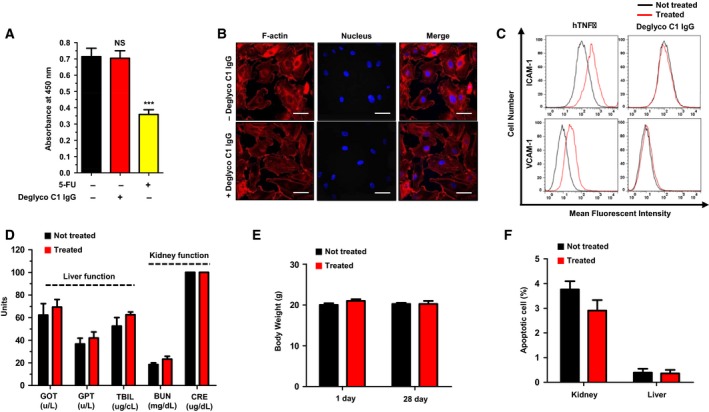
Evaluation of the *in vitro* and *in vivo* toxicity of the optimized lead antibody. (A) HUVECs were incubated in the absence (MOCK) or presence of deglyco C1 IgG or 5‐FU (positive control) for 2 days. Cell viability was assessed by measuring absorbance at 450 nm. Values represent the mean ± SEM of triplicate measurements from two independent experiments. ***P <* *0.001. (B) HUVECs cultured in the presence or absence of deglyco C1 IgG were stained with rhodamine–phalloidin and 4′, 6‐diamidino‐2‐phenylindole, dihydrochloride, and cell morphologies were examined by confocal microscopy. Scale bars = 20 μm. (C) HUVECs were cultured in the presence or absence of hTNFα or deglyco C1 IgG, stained with anti‐ICAM‐1 (upper panel) or VCAM‐1 (lower panel) polyclonal antibody, and analyzed by flow cytometry. hTNFα served as the positive control for endothelial cell activation. Results are representative of three independent experiments. (D) *In vivo* toxicity was detected based on changes in the serum concentrations of GOT, GPT, blood urea nitrogen, creatinine, and TBIL measured 30 days after antibody injection. (E) *In vivo* toxicity was also detected based on changes in the body weights of mice between day 1 and day 28 after antibody injection. (F) The apoptotic status of kidney and liver tissues 30 days after antibody injection was analyzed by TUNEL assay. All of the data represent the mean ± SD from four independent experiments.

To investigate the effect of deglyco C1 IgG on endothelial cell viability, we treated HUVECs with the apoptotic inducer 5‐fluorouracil (5‐FU) in the presence or absence of deglyco C1 IgG. Deglyco C1 IgG alone exerted no cytotoxic effects on the HUVECs, whereas 5‐FU significantly reduced their viability (Fig. 4A). To further examine the effects of deglyco C1 IgG on endothelial cells, we evaluated the morphological changes of HUVECs in response to deglyco C1 IgG by staining F‐actin structures with rhodamine–phalloidin. We found no cytotoxicity‐related changes in cell morphology upon treatment with deglyco C1 IgG (Fig. 4B). Next, we investigated the effect of deglyco C1 IgG on endothelial cell activation, which occurs in response to harmful stimuli and can be monitored by expression of the endothelial cell activation markers VCAM‐1 and ICAM‐1. Human tumor necrosis factor‐alpha (hTNFα) treatment was used as a positive control. Deglyco C1 IgG had little effect on HUVEC activation, whereas hTNFα induced activation of the HUVECs (Fig. 4C). Collectively, these data suggest that deglyco C1 IgG does not cause significant endothelial cell toxicity.

To evaluate the *in vivo* toxicity of deglyco C1 IgG, we intravenously injected deglyco C1 IgG twice a week into normal mice and then compared the liver and kidney function, body weight, and apoptotic status of the liver and kidney tissues in the control and treated groups. Liver function was determined by measuring serum concentrations of GOT, GPT, and TBIL, and kidney function was determined by measuring BUN and CRE concentrations. Apoptosis was measured by TUNEL staining. No significant changes in liver function, kidney function (Fig. 4D), body weight (Fig. 4E), or apoptosis status (Fig. 4F) were observed. These results suggest that deglyco C1 IgG does not induce severe toxicity *in vivo*.

### 3.5 Effect of the optimized lead antibody on VEGF‐dependent angiogenesis

**Figure 5:**
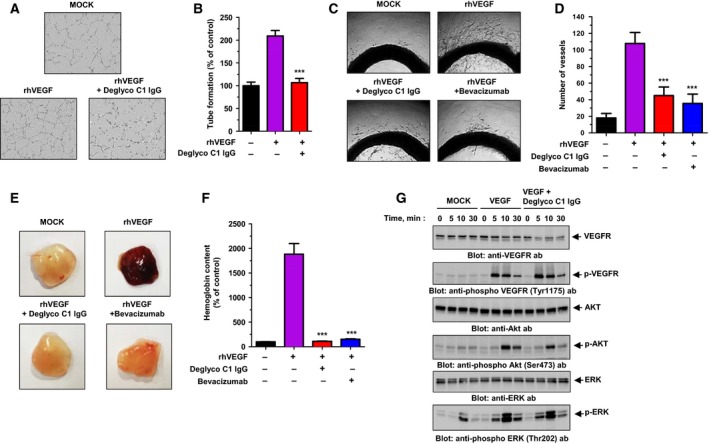
Effect of the optimized lead antibody on VEGF‐dependent angiogenesis. (A) rhVEGF‐dependent tube formation was assessed in the absence (MOCK) or presence of deglyco C1 IgG or bevacizumab. (B) Total branch numbers were expressed as a percent of tube formation in the control (MOCK). (C) Images of rhVEGF‐dependent vessel sprouting were obtained from sectioned aortic rings cultured in the absence (MOCK) or presence of deglyco C1 IgG or bevacizumab. (D) The numbers of sprouting vessels were counted manually. (E) Nude mice were implanted with Matrigel plugs in the absence (MOCK) or presence of deglyco C1 IgG or bevacizumab, and images of rhVEGF‐dependent microvessel formation were obtained. (F) The extent of microvessel formation was determined by measuring the hemoglobin content, which was expressed as a percent of the hemoglobin content in the control (MOCK). All of the values represent the mean ± SEM of triplicate measurements from two independent experiments. ***P <* *0.001. (G) Immunoblot analysis was performed to assess rhVEGF‐dependent phosphorylation of VEGFR, Akt, and ERK in HUVECs in the absence (MOCK) or presence of VEGF or VEGF plus deglyco C1 IgG.

To investigate the effect of deglyco C1 IgG on VEGF‐dependent angiogenesis *in vitro*, we treated HUVECs with rhVEGF in the presence or absence of deglyco C1 IgG and evaluated HUVEC tube formation. Deglyco C1 IgG abrogated completely rhVEGF‐dependent tube formation (Fig. 5A,B). We then performed *ex vivo* rat aortic ring assays in the presence or absence of deglyco C1 IgG. Few vessels sprouted from rat aortas in EBM, whereas abundant vessels sprouted in the presence of rhVEGF. Notably, the addition of deglyco C1 IgG significantly reduced the number of vessels that sprouted from rat aortas in the presence of rhVEGF (Fig. 5C,D). To evaluate the effect of deglyco C1 IgG on VEGF‐dependent angiogenesis *in vivo*, we analyzed microvessel formation in a mouse Matrigel model using plug assays and measuring hemoglobin levels as indicators of microvessel formation. Deglyco C1 IgG almost completely blocked the increase in hemoglobin observed in the presence of rhVEGF (Fig. 5E,F).

To examine the effect of deglyco C1 IgG on VEGF‐dependent signaling in endothelial cells, we performed immunoblot analysis. rhVEGF‐treated HUVECs were grown in the presence or absence of deglyco C1 IgG, and changes in the phosphorylation of VEGF signaling molecules, including VEGF receptor (VEGFR), Akt, and ERK, were monitored. Deglyco C1 IgG had little effect on VEGF‐dependent phosphorylation of VEGFR, Akt, or ERK in HUVECs (Fig. 5G). Collectively, these data suggest that deglyco C1 IgG may significantly suppress abnormal VEGF‐dependent angiogenesis *in vivo* without adversely affecting VEGF‐mediated signaling in normal endothelial cells.

### 3.6 Effect of the optimized lead antibody on tumor angiogenesis

**Figure 6:**
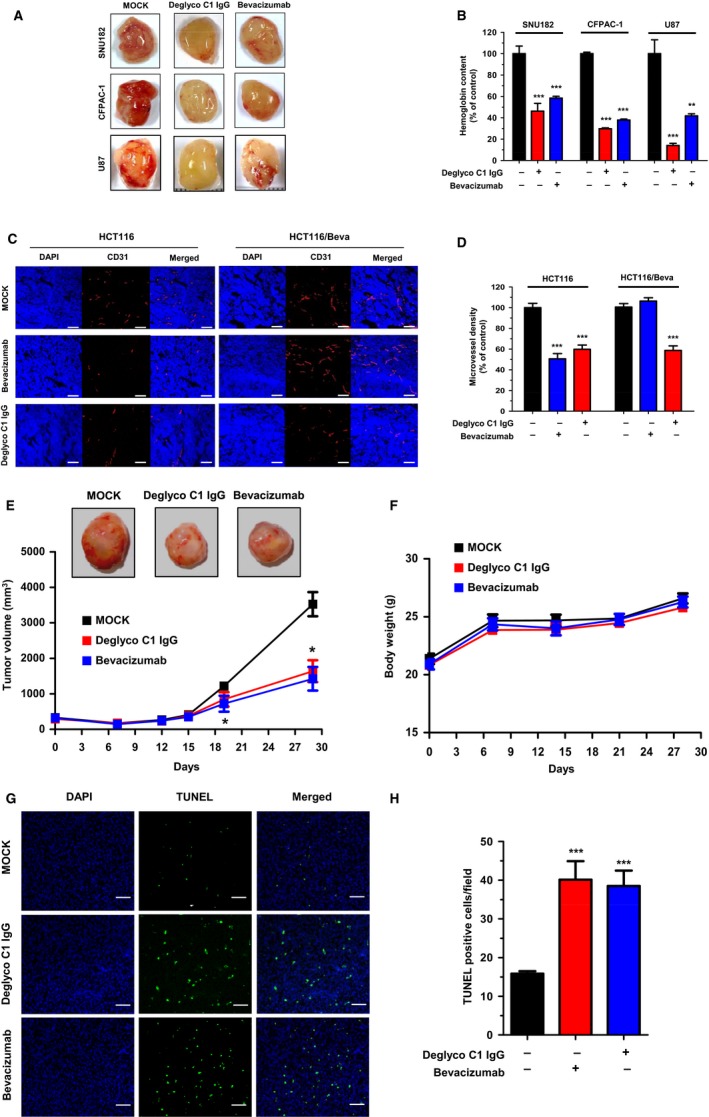
Effect of the optimized lead antibody on tumor angiogenesis. (A) Microvessel formation by SNU182, CFPAC‐1, and U87 cell‐derived tumors was measured in the absence (MOCK) or presence of deglyco C1 IgG or bevacizumab. (B) Hemoglobin content was expressed as a percent of the hemoglobin content in the control (MOCK). (C) Microvessel formation by HCT116 and HCT116/Beva cell‐derived tumors in the absence (MOCK) or presence of deglyco C1 IgG or bevacizumab was determined by immunohistochemistry with anti‐CD31 antibody. Scale bars = 100 μm. (D) CD31 positivity per field was expressed as a percent of the CD31‐positive microvessel density in the control (MOCK). All of the values represent the mean ± SEM of quadruplicate measurements from two independent experiments. BALB/c male nude mice were subcutaneously implanted with U87 glioma cell‐loaded Matrigel plugs, with or without 10 mg·kg^−1^ deglyco C1 IgG or bevacizumab. Tumor volume (E) and total body weight (F) were measured once a week for 1 month. Tumor images at 1 month after injection are also shown (*inset*). All of the values represent mean ± SEM. (G) Apoptotic tumor cells were determined by TUNEL staining in control (MOCK) group and deglyco C1 IgG‐ or bevacizumab‐treated groups. Scale bars = 50 μm. (H) TUNEL positivity per field in each group was expressed as a graph bar. All of the values represent the mean ± SEM of quadruplicate measurements. *P <* *0.05 and ***P <* *0.001.

To investigate the effect of deglyco C1 IgG on tumor angiogenesis, we conducted tumor cell‐loaded Matrigel plug angiogenesis assays in athymic nude mice. SNU182 human hepatocellular carcinoma cell‐, CFPAC‐1 human pancreatic cancer cell‐, and U87 human glioma cell‐loaded Matrigel plugs were implanted into athymic nude mice with or without a single 10 mg·kg^−1^ dose of deglyco C1 IgG. After 2 weeks, the Matrigel plugs were removed and the total hemoglobin content in each plug was measured spectrophotometrically. We found that deglyco C1 IgG significantly reduced the increase in hemoglobin content, which was triggered by SNU182, CFPAC‐1, and U87 cell‐loaded plugs (Fig. 6A,B), indicating that deglyco C1 IgG had an anti‐angiogenic effect on tumor angiogenesis.

To further examine the effect of deglyco C1 IgG on tumor angiogenesis, we performed Matrigel plug angiogenesis assays with HCT116 human colorectal cancer cells (HCT116) and bevacizumab‐adapted HCT116 cells (HCT116/Beva) in the presence or absence of a single 5 mg·kg^−1^ dose of deglyco C1 IgG or bevacizumab. Following a period of 14 days, the Matrigel plugs were removed and subjected to immunohistochemistry for CD31, a marker of microvessels. Microvessel density was determined by quantifying CD31 positivity per field from each image acquired by confocal microscopy. We found that deglyco C1 IgG or bevacizumab similarly and significantly reduced microvessel formation in HCT116 cells. Microvessel formation in HCT116/Beva cells was significantly inhibited by deglyco C1 IgG, but not by bevacizumab (Fig. 6C,D) indicating the superior ability of deglyco C1 IgG to inhibit tumor angiogenesis in bevacizumab‐resistant colorectal cancer cells.

To check the effect of deglyco C1 IgG on tumor growth and apoptosis, we subcutaneously injected U87 human glioma cell‐loaded Matrigel plugs into athymic nude mice with or without a single 10 mg·kg^−1^ dose of deglyco C1 IgG or bevacizumab and monitored tumor size and total body weight until 1 month. We found that deglyco C1 IgG significantly reduced the tumor size to a similar extent of bevacizumab without affecting body weight (Fig. 6E,F). Then, model mice were sacrificed at 2 weeks after injection and xenograft tumors were subjected to immunohistochemical examination. Apoptotic tumor cells were determined by TUNEL staining. We found that more apoptoses of tumor cells were displayed in deglyco C1 IgG‐ or bevacizumab‐treated groups than in control group (Fig. 6G,H). Collectively, these results suggest that deglyco C1 IgG has the potential to suppress tumor angiogenesis *in vivo*.

## 4 Discussion

Antibody optimization is a key challenge for the successful development of therapeutic antibodies, and a poor understanding of complex antibody structural conformations combined with technical limitations has hindered our ability to improve the attributes of antibodies for use as therapeutic drugs. In this study, we successfully generated a novel anti‐angiogenic human monoclonal antibody that targets CLEC14a‐CTLD and exhibits improved stability and homogeneity compared to the parental antibody. This is the first study to propose use of an optimized antibody targeting CLEC14a‐CTLD, and the first study to demonstrate that CLEC14a‐CTLD is a novel anti‐angiogenic target for VEGF‐dependent angiogenesis and tumor angiogenesis.

Monoclonal antibody aggregation is affected by various factors, including freeze‐thawing, temperature, and the physicochemical reactions that occur during antibody development and production (Wang *et al*., 2007). Antibody aggregation is believed to induce the immunogenicity of antibody therapeutics and can limit large‐scale antibody production (Bessa *et al*., 2015; Joubert *et al*., 2012; Moussa *et al*., 2016; Ratanji *et al*., 2014; Rombach‐Riegraf *et al*., 2014). Thus, an antibody must be optimized and its aggregation minimized before pharmaceutical‐grade antibodies can be produced. The CLEC14a‐CTLD parental antibody showed significant anti‐angiogenic activity *in vitro* (Ki *et al*., 2013), but approximately 95% of the antibodies formed visible aggregates during purification. Thus, in this study, we performed CDR grafting to improve antibody stability. We obtained IgG clones 1–4, and our optimization strategy enabled us to select Clone 1 IgG as the most stable antibody platform. Our comparative analysis indicated that CDR grafting essentially eliminated the visible aggregation of Clone 1 IgG. Clones 1–4 expressed similar amounts of total antibody, whereas clones 2–4 yielded much smaller amounts of soluble antibody than Clone 1 following centrifugation (data not shown), which supports the effectiveness of our strategy.

*N*‐glycosylation is a post‐translational protein modification that often occurs in antibody CDR regions (Abel *et al*., 1968; Dunn‐Walters *et al*., 2000) and can adversely affect the quality, safety, and efficacy of an antibody (Coloma *et al*., 1999; Igawa *et al*., 2011). Therefore, we used a deglycosylation procedure to remove a putative *N*‐glycosylation consensus sequence (amino acids 32–34, NNS) from the CDR1 region of Clone 1 IgG *V*_L_. We isolated deglycosylated antibody clones that bound to human and mouse CLEC14a‐CTLD, and we used consecutive functional assays to select deglyco C1 IgG as a homogeneity‐improved antibody with potent anti‐angiogenic activity comparable to the anti‐angiogenic activity of bevacizumab.

C‐type lectin‐like 14a is known to be an endothelial cell‐specific adhesion molecule that mediates endothelial cell–cell contacts during angiogenesis (Ki *et al*., 2013; Rho *et al*., 2011). Although this is thought to involve the CTLD, no clear evidence has emerged to date showing that CLEC14a‐CTLD is a key domain for the interaction between CLEC14a molecules. Our finding that deglyco C1 IgG inhibited the interaction between CLEC14‐CTLD and CLEC14a‐ECD leads us to speculate that CLEC14a‐CTLD may participate in the interaction between CLEC14a molecules and that the optimized antibody targeting CLEC14a‐CTLD blocks this interaction by inhibiting the CTLD‐mediated interaction between CLEC14a molecules on adjacent endothelial cells during angiogenesis. Furthermore, we confirmed that the deglyco C1 IgG antibody could concomitantly downregulate expression of CLEC14a on the surface of endothelial cells. Taken together, these findings suggest that CLEC14a‐CTLD may be a key domain involved in the regulation of CLEC14a‐mediated angiogenesis.

A number of lines of evidence support the idea that CLEC14a‐CTLD may be a novel anti‐angiogenic target for VEGF‐dependent angiogenesis and tumor angiogenesis. First, CLEC14a is a tumor endothelial marker protein that is exclusively expressed on tumor vessels, but not on normal vessels during tumor angiogenesis (Mura *et al*., 2012). In addition, deglyco C1 IgG was found to bind to a specific epitope of CLEC14a‐CTLD (Fig. S3) with nanomolar affinity and cross‐species reactivity to human and mouse CLEC14a. Our *in vitro* and *in vivo* efficacy tests showed that deglyco C1 IgG significantly inhibits VEGF‐dependent angiogenesis. Notably, deglyco C1 IgG significantly inhibited tumor angiogenesis in four different angiogenesis animal models, including SUN152 human hepatocellular carcinoma, CFPAC‐1 human pancreatic cancer, U87 human glioma, and HCT116 and HCT116/Beva human colorectal cell lines. Furthermore, we confirmed that deglyco C1 IgG significantly reduced the tumor size and increased the apoptosis of U87 cells to a similar degree as bevacizumab without affecting body weight (Fig. 6E‐H). We found that deglyco C1 IgG did not significantly affect the viability, morphology, or activation of HUVECs *in vitro* suggesting that the optimized antibody may not cause significant endothelial toxicity *in vivo*. In addition, we demonstrated that deglyco C1 IgG did not significantly affect kidney and liver functions, body weight, or cell viability indicating that it does not induce severe toxicity *in vivo*.

Under pathological conditions, high levels of VEGF lead to abnormal angiogenesis that is closely associated with the progression and metastasis of various cancers (Goel and Mercurio, 2013). Bevacizumab is a highly successful anti‐angiogenic humanized antibody that targets VEGF and that is widely used in clinical cancer treatments. However, the clinical usefulness of bevacizumab is limited by significant side effects (Gordon and Cunningham, 2005; Taugourdeau‐Raymond *et al*., 2012) and drug resistance (Giuliano and Pages, 2013; Kopetz *et al*., 2010; Labussiere *et al*., 2016). Therefore, the development of additional specific antibody therapeutics is crucial. Based on our results, we suggest that our optimized antibody may have therapeutic potential. For example, deglyco C1 IgG significantly inhibited VEGF‐dependent vascular formation without affecting the phosphorylation of the VEGF‐mediated signaling molecules VEGFR, Akt, and ERK. Our combined findings suggest that this improved antibody may effectively suppress abnormal VEGF‐dependent angiogenesis by CLEC14a‐positive tumor vessels without affecting VEGF signaling in CLEC14a‐negative normal endothelial cells. Furthermore, the finding that deglyco C1 significantly inhibited tumor angiogenesis triggered by HCT116/Beva cells, while bevacizumab IgG had no effect, suggests that the optimized antibody may be useful for effectively inhibiting bevacizumab‐resistant tumor angiogenesis. Lastly, our observation that deglyco C1 IgG potently inhibits both EGM‐dependent angiogenesis and VEGF‐dependent angiogenesis further indicates that the optimized antibody may be widely useful for inhibiting angiogenesis induced by a variety of angiogenic factors. Collectively, these findings suggest that our optimized antibody may be a useful alternative and/or adjuvant therapy to efficiently suppress VEGF‐dependent and tumor angiogenesis.

## 5 Conclusions

In conclusion, in this study, we described an optimized antibody, deglyco C1 IgG, that targets CLEC14a‐CTLD and demonstrated that targeting of CLEC14a‐CTLD may be an effective strategy for suppressing CLEC14a‐mediated VEGF‐dependent and tumor angiogenesis, which is closely associated with pathological angiogenesis. Our evidence also suggests that CLEC14a‐CTLD may be a novel potential anti‐angiogenic target for VEGF‐dependent angiogenesis and tumor angiogenesis. In future studies, we plan to evaluate the *in vivo* efficacy of deglyco C1 IgG in greater depth using angiogenesis‐related disease models.

## Author contributions

TK, CSP, JJ, MRK, ESK, SJK, HJK, and KL designed and performed the experiments; BCY, HN, JL, DYK, KC, TGL, HK, and YK analyzed and interpreted the data. SL obtained funding, conceptualized, designed, and supervised the study, and wrote the manuscript. All of the authors reviewed and approved the final manuscript.

## Supporting information


**Fig. S1.** Biochemical analysis of Clone 1 and deglyco C1 scFv by two‐dimensional gel electrophoresis.
**Fig. S2.** Biochemical characterization of the optimized lead antibody.
**Fig. S3.** Identification of the CLEC14a‐CTLD epitope for deglyco C1 IgG.
**Table S1.** Summary of theoretical stability of parental IgG and CDR‐grafted IgGs.
