# Supplementary material for: Inhibition of VEGF‐dependent angiogenesis and tumor angiogenesis by an optimized antibody targeting CLEC14a
Source: Mol Oncol. 2018 Jan 26;12(3):356–72. doi: 10.1002/1878-0261.12169 (PMC5830631; doi:10.1002/1878-0261.12169)
Supplement: Supplementary file 1 — Fig. S1. Biochemical analysis of Clone 1 and deglyco C1 scFv by two‐dimensional gel electrophoresis. Fig. S2. Biochemical characterization of the optimized lead antibody. Fig. S3. Identification of the CLEC14a‐CTLD epitope for deglyco C1 IgG. Table S1. Summary of theoretical stability of parental IgG and CDR‐grafted IgGs. [file MOL2-12-356-s001.pdf]

Table. S1. Summary of theoretical stability of parental IgG and CDR-grafted IgGs

Antibodies	Rank	Developability Index (DI)
Clone 1 IgG	1	156.13
Clone 2 IgG	2	170.17
Clone 3 IgG	3	181.75
Clone 4 IgG	4	182.46
Parental IgG	5	244.20

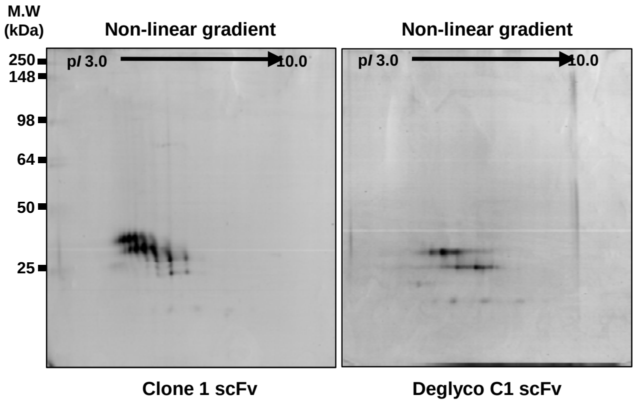

Fig. S1. Kim et al.

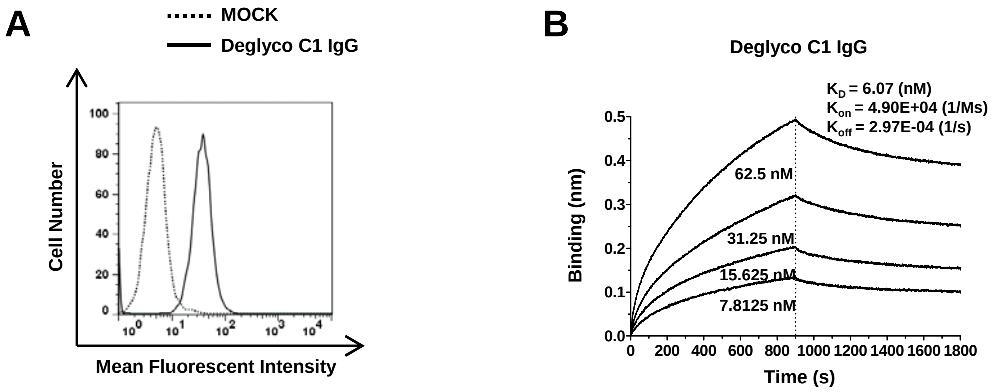

Fig. S2. Kim et al.

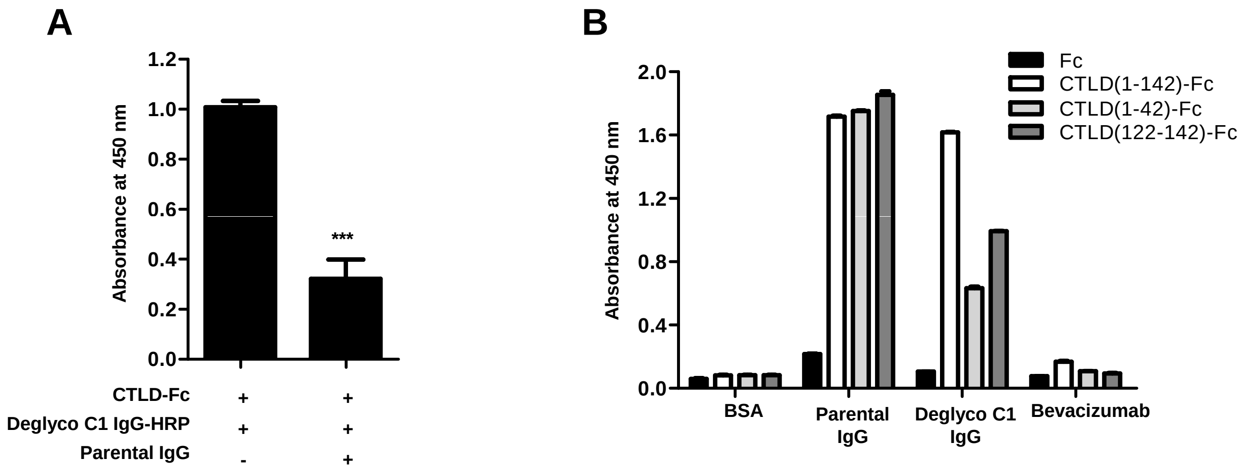

Fig. S3. Kim et al.
